# Prevalence and Associated Factors of Perinatal Asphyxia among Neonates in General Hospitals of Tigray, Ethiopia, 2018

**DOI:** 10.1155/2018/5351010

**Published:** 2018-11-01

**Authors:** Gdiom Gebreheat, Tesfay Tsegay, Dessalegn Kiros, Hirut Teame, Natnael Etsay, Guesh Welu, Desta Abraha

**Affiliations:** ^1^Department of Nursing, College of Medicine and Health Sciences, Adigrat University, Adigrat, Ethiopia; ^2^Department of Public Health, College of Medicine and Health Sciences, Adigrat University, Adigrat, Ethiopia; ^3^Department of Midwifery, College of Medicine and Health Sciences, Adigrat University, Adigrat, Ethiopia

## Abstract

Perinatal asphyxia is one of the most important causes of morbidity and mortality in neonates. Perinatal asphyxia occurs in association with maternal, fetal, and maternofetal factors. However, the magnitude and associated factors of perinatal asphyxia are not well studied in Tigray, Ethiopia. Therefore, our study is conducted to determine the prevalence and factors associated with perinatal asphyxia among neonates in general hospitals of Tigray. An observational hospital-based cross-sectional study was conducted in randomly selected general hospitals. A semistructured questionnaire was used to collect data from 421 randomly selected neonates with their mothers and medical records. The data was entered into epidata version 3.5 and exported to Statistical Package for Social Sciences (SPSS) version 20 for analysis. Finally, the presence of an association between a dependent variable and an independent variables has been declared at P-value ≤0.05, or adjusted odds ratio (AOR), 95% confidence interval (CI). Accordingly, the result of this study showed that the prevalence of perinatal asphyxia among the selected general hospitals was 22.1%. Neonates born with cesarean section are seven times more likely to have perinatal asphyxia than those who are born spontaneously through the vagina (AOR, 6.97; CI (2.87-16.93)). In addition, neonates who are born meconium stained are 8.55 times more likely to have perinatal asphyxia than those who had not stained with meconium (AOR, 8.55; CI (4.20-17.39)). Neonates who are weighed less than 2.5 kg are 12.75 times more likely to have perinatal asphyxia than those who are weighed 2.5-4 kg (AOR, 12.75; CI (4.05-40.08)). Prolonged duration of labour was also associated statistically with perinatal asphyxia (AOR, 3.33, CI (1.32-8.38)). In conclusion, the magnitude of perinatal asphyxia in general hospitals of Tigray remains high. Low birth weight, meconium-stained amniotic fluid, cesarean section, and prolonged maternal labour have been associated with perinatal asphyxia.

## 1. Introduction

Perinatal asphyxia is defined as an oxygen deprivation that occurs around the time of birth and may be caused by several perinatal events. It is also stated as evolution from the utilization of a single indicator such as low Apgar (Appearance, Pulse, Grimace, Activity, and Respiration) score or delayed respiration to a multiple indicators approaches focusing especially on the neurological damage. The neonatal period is the first 28 days of life but also the most vulnerable time for survival. Globally, 45% under-five children death occurs during the neonatal period [1–4]. About one-quarter of all neonatal deaths are caused by perinatal asphyxia in worldwide. Perinatal asphyxia is responsible for 23% neonatal deaths in low-income countries. This finding underlines that perinatal asphyxia is still a burden of the world [1, 2, 5].

Various factors are associated with the development of perinatal asphyxia. An institutional based cross-sectional study in Pakistan revealed that instrumental delivery (n=46), spontaneous vertex delivery, cesarean section, prolonged rupture of membranes, meconium staining, maternal fever, and anaemia at delivery were significantly associated with birth asphyxia [6]. According to the Ethiopia Health Demography Survey (EDHS), among the direct causes of under-five mortality, asphyxia was accounted for 14% of the death [7]. However, in Tigray there is no study found to assess issues on prevalence and associated factors of perinatal asphyxia. Therefore, the main purpose of this study is to explore and address the gap in prevalence and associated factors of perinatal asphyxia in Tigray general hospitals. Moreover, it will have greater input to program managers and policy-makers in designing, proper implementation, and evaluation of programs on the reduction of under-five children mortality and improvement of children health care.

## 2. Methods and Materials

This is an observational hospital-based cross-sectional study conducted in Tigray region, Ethiopia, from December 1, 2017, to February 1, 2018. In this region, there are 14 general hospitals. All newborns with their mothers in the delivery units of General Hospitals of Tigray were the source of population. The Apgar score provides a convenient shorthand for reporting the status of the newborn infant and to initiate resuscitation, especially in resource-limited settings. However, the Apgar score has a limited time frame and includes subjective components. In addition, evidence of biochemical markers (metabolic acidosis and multiorgan failure) is significant before the score is affected to diagnose perinatal asphyxia. Components of the score such as tone, colour, and reflex partially depend on the physiologic maturity of the neonate [8]. To minimize such limitations of Apgar score for perinatal asphyxia diagnosis, neonates who are suffering from major congenital anomalies or syndromes and preterm babies <35 completed weeks were excluded from the study. In addition, neonates whose first minute Apgar score <7 were considered as having perinatal asphyxia. Sample size was calculated using a single population proportion formula and the following assumptions were considered: proportion of perinatal asphyxia and/or its associated factors 50% (p=0.5), level of significance to be 5% (*α* = 0.05), 95 % confidence level (Z*α*/2 = 1.96), and absolute precision or margin of error to be 5% (d = 0.05). Based on this, adding 10% nonresponsive rate, the final sample size required for this study was 422 neonates.

### 2.1. Sampling Procedure

There are 14 general Hospitals in Tigray region. Of those, 6 general hospitals are randomly selected for this study, namely, Kahsay Abera General Hospital, Suhul General Hospital, St. Mary Axum General Hospital, Adigrat General Hospital, Mekelle General Hospital, and Lemlem Karl General Hospital. The number of study participants for each hospital was estimated proportionally based on their monthly average number of delivery in each hospital. A systematic sampling technique was used to get all study units in the delivery unit of the hospitals. In this study, all neonates with their mother were eligible to participate while neonates who are suffering from major congenital anomalies or syndromes and preterm babies <35 completed weeks were excluded.

### 2.2. Data Collection Procedure

Data were collected by using semistructured questionnaire from primary and secondary (chart review) sources. This questionnaire is adapted from various related studies [9–12]. A pretested structured interviewer based questionnaire was used to collect data on maternal sociodemographic profiles, such as age and educational status. Data related to antepartum (such as parity, antepartum hemorrhage, and antenatal visits), intrapartum (such as fetal presentation, mode of delivery, meconium-stained amniotic fluid, and premature rupture of membranes), and neonatal factors (such as gestational age, birth weight, and sex) were abstracted using a pretested structured checklist from the medical records of the neonates and their mothers. Data collection was conducted with a trained BSc holder midwife and supervised with MSc holder nurse in each general Hospital.

### 2.3. Data Analysis

The collected data were checked manually for completeness and then coded and entered into Epi data version 3.5 and exported to SPSS version 20. After data exploration and cleaning a univariate analysis was done using frequency and per cent. Using bivariate analysis candidate variables were identified for multiple regressions at a p-value of 0.05. Again, those variables showed significant association on bivariate analyses were entered into multiple logistic analyses to control (adjust) possible confounding variable and to identify independent predictors variable. Finally, multivariate analysis was used to declare the presence of an association between a dependent variable and an independent variables at a p-value less than 0.05, or AOR, 95% CI.

### 2.4. Ethical Consideration

Ethical clearance and approval were obtained from the research and community service directorate of Adigrat University with a code number of ADU/CMHS/032/09. From Tigray health bureau official letters were taken to each general Hospital in Tigray. Prior to data collection, the objective of the study was explained to mothers. Above all, the confidentiality of the study participants was kept.

## 3. Result

### 3.1. Profile of the Study Participants

A total of 421 neonates with their mothers were involved in the study with a response rate of 99.7%. Majority 396(94.1%) of the neonates born at ≥37weeks of gestational age and nearly half 209(49.6%) of them were females while all the neonates mean birth weight is 3.16 kg. The median age of the mothers was 27 years with the majority of 240(57%) were in the range of 25-34. Out of these 421 neonates, 93(22.1%) were cases of perinatal asphyxia and the rest 328(77.9%) had no perinatal asphyxia based on the APGAR score of less than 7 at first minute of delivery. Among the cases with perinatal asphyxia male to female ratio was 1.07:1.

### 3.2. Antepartum Associated Factors of Perinatal Asphyxia

On bivariate regression, the crude odds ratios (COR) at (95% CI) for perinatal asphyxia were maternal illiteracy 1.81(1.11-2.97), ANC (Antenatal care) follow-up 3.67(1.04-12.96), and maternal comorbidity during pregnancy such as anaemia 7.11(3.13-16.15), preeclampsia 4.68(2.28-9.60), and antepartum haemorrhage 5.71(2.80-11.70). However, maternal age and parity status did not appear to contribute to perinatal asphyxia (Table 1).

### 3.3. Intrapartum and Neonatal Associated Factors of Perinatal Asphyxia

On bivariate analysis, maternal conditions during labour and delivery such as prolonged labour 8.78(4.81-16.02), cephalopelvic disproportion (CPD) 10.32(3.88-27.45), and peripartum pyrexia 5.36(2.09-13.77) were significantly associated with perinatal asphyxia. On the other side, neonatal, nonvertical presentation 5.979(3.11-11.47), prematurity 6.46(2.28-18.30), and weight less than 2.5 kg 6.89 (3.00-15.81) had been associated with perinatal asphyxia at COR (95% CI) while their gestational age is not (Table 2).

### 3.4. Factors Associated with Perinatal Asphyxia

Variables showed significant association on bivariate analyses at a p-value of 0.05 that has been entered into multiple logistic analyses to control (adjust) possible confounding variables. Based on this, mode of delivery, prolonged labour, meconium stained, and neonatal weight had a significant association with perinatal asphyxia at AOR (95% CI). Neonates born with cesarean section are seven times more likely to have perinatal asphyxia than those who are born spontaneously through the vagina 6.97(2.87-16.93). In addition, those who are weighed less than 2.5 kg are 12.75 times more likely to have perinatal asphyxia than those who are weighed 2.5-4kg 12.75(4.05-40.08). Prolonged duration of labour was also associated statistically with perinatal asphyxia 3.33(1.32-8.38). Neonates who are born meconium stained are 8.55 times more likely to have perinatal asphyxia than those who had not stained with meconium 8.55(4.20-17.39) (Table 3).

## 4. Discussion

The prevalence of perinatal asphyxia among the selected general hospitals was 22.1% though the ANC coverage was 411(97.6%). This figure is very high when compared with prevalence of 0.24% in Canada, 0.85% in the Netherlands, 2.5% observed in Dire Dawa, Ethiopia, and 2.69 in Nepal but lower than the study in Nigeria which was 29.4% [10, 13–16]. In addition, male to female ratio was found to be 1.07:1 which is similar to study in India [17]. This difference could be due to the level of quality care provided during antenatal, natal, or postnatal period among nations. Moreover, our study has also revealed that the maternal and neonatal factors associated with perinatal asphyxia. Among the identified factors, prolonged labour was found significantly higher in the asphyxiated neonates. This finding is similar to studies in Ethiopia, Cameroon, Colombia, and Pakistan [10, 18–20]. Regarding mode of delivery, 65(15.4%) neonates were delivered via caesarean section which is consistent with the WHO(world health organization) recommended rate, 10-15%, but lower than the studies in Turkey, China, Nepal, India, and Cameroon which was 38.1%, 42%, 22.1%, 25%, and 45.6%, respectively [12, 18, 21–24]. It might be due to cesarean section delivery promotions while mothers have no indications, in some nations. Caesarean section was also strongly associated with birth asphyxia in comparison to vaginal delivery and various studies supported it with different study designs [12, 18, 24]. The risk of asphyxia may lower as the fetal chest passes through the birth canal (vagina) is compressed, squeezing excess fluid out of the lungs, which would be a risk for asphyxia. Another contributing factor for perinatal asphyxia was neonatal birth weight. Neonates who are weighed less than 2.5 kg were prone to develop birth asphyxia which is consistent with studies from other corners of the world [9, 11]. In contrary, a large size of a baby at birth (a proxy for birth weight) had been found an associated factor for birth asphyxia-related neonatal mortality [20]. Meconium-stained amniotic fluid also found significantly associated with the incidence of perinatal asphyxia like other studies indicated it before [9, 11, 17, 25]. Presence of meconium in the amniotic fluid may lead to aspiration of it into the lung that could eventually result in perinatal asphyxia.

## 5. Conclusion

Perinatal asphyxia is one of the common causes of neonatal morbidity and mortality in neonatal intensive care unit (NICU). In spite of advances in the management of the various neonatal problems in the delivery department, the prevalence of perinatal asphyxia is 22.1%. Low birth weight, meconium-stained amniotic fluid, caesarean section, and prolonged maternal labour were the factors associated with perinatal asphyxia.

## Figures and Tables

**Table 1:** Bivariate regression on antepartum associated factors of perinatal asphyxia among neonates in general hospitals of Tigray, Ethiopia, 2018.

**Variable**	**No Perinatal asphyxia **	**Perinatal asphyxia **	**COR (95**%**)**	**p-value**
**Maternal age**				
15-24	105	24	0.80(0.47-1.38)	0.433
25-34	187	53	1	
35-49	36	16	1.57(0.81-3.04)	0.184

**Educational status**				
Illiterate	79	34	1.81(1.11-2.97)	0.017
Literate	249	59	1	

**Parity status of the mother**				
Primigravida	127	38	1.09(0.68-1.75)	0.71
Multigravida	201	55	1	

**ANC follow up**				
Yes	323	88	1	
No	5	5	3.67(1.04-12.96	0.043

**History of abortion**				
Yes	65	25	1.49(0.87-2.53)	0.144
No	263	68	1	

**Maternal comorbidity during pregnancy **				
Hypertension				
Yes	5	7	5.26(1.63-16.97)	0.006
No	323	86	1	

**Anaemia **				
Yes	10	17	7.11(3.13-16.15)	0
No	318	76	1	

**Preeclampsia **				
Yes	16	18	4.68(2.28-9.60)	0
No	312	75	1	

**Eclampsia**				
Yes	8	9	4.29(1.60-11.44)	0.004
No	320	84	1	

**Antepartum haemorrhage **				
Yes	15	20	5.71(2.80-11.70)	0
No	313	73	1	

**Table 2:** Bivariate regression on intrapartum and neonatal associated factors of perinatal asphyxia among neonates in general hospitals of Tigray, Ethiopia, 2018.

**Variable**	**No Perinatal asphyxia **	**Perinatal asphyxia **	**COR (95**%**)**	**p-value**
**Fetal presentation **				
Vertex	309	68	1	
Non-vertex	19	25	5.979(3.11-11.47)	0

**Mode of delivery**				
Vaginal	304	52	1	
cesarean section	24	41	9.98(5.57-17.90)	0

**If vaginal **				
Spontaneous	284	46	1	
Assisted	20	7	2.16(0.865-5.39)	0.099

**Maternal conditions during labour and delivery**				

**Premature rupture of membrane**				
Yes	26	19	2.98(1.56-5.67)	0.001
No	302	74	1	

**Prolonged labour**				
Yes	22	36	8.78(4.81-16.02)	0
No	306	57	1	

**Cephalopelvic disproportion**				
Yes	6	15	10.32(3.88-27.45)	0
No	322	78	1	

**Peripartum pyrexia **				
Yes	8	11	5.36(2.09-13.77)	0
No	320	82	1	

**Neonatal conditions after delivery **				

**Meconium-stained **				
Yes	39	55	10.72(6.30-18.25)	0
No	289	38	1	

**Prolapsed cord**				
Yes	10	11	4.26(1.75-10.39)	0.001
No	318	82	1	

**Prematurity **				
Yes	6	10	6.46(2.28-18.30)	0
No	322	83	1	

**Gestational age**				
<37weeks	17	8	1.72(0.72-4.12)	0.223
≥37 weeks	311	85	1	

**Sex of neonate**				
Male	164	48	1.06(0.67-1.69)	0.784
Female	164	45	1	

**Weight of neonate**				
<2.5kg	10	16	6.89(3.00-15.81)	0
2.5-4kg	310	72	1	
>4kg	8	5	2.69(0.85-8.46)	0.091

**Table 3:** Multivariate regression on factors associated with perinatal asphyxia among neonates in general hospitals of Tigray, Ethiopia, 2018.

**Variable**	**No Perinatal asphyxia **	**Perinatal asphyxia **	**AOR (95**%**)**	**p-value**
**Educational status**				
Illiterate	79	34	1.46(0.73-2.94)	0.281
Literate	249	59	1	

**ANC follow up**				
Yes	323	88	1	
No	5	5	3.69(0.65-20.80)	0.139

**Maternal comorbidity during pregnancy **				

**Hypertension **				
Yes	5	7	1.79(0.25-12.79)	0.56
No	323	86	1	

**Anaemia **				
Yes	10	17	3.41(0.79-14.64)	0.099
No	318	76	1	

**Preeclampsia **				
Yes	16	18	2.64(0.96-7.29)	0.06
No	312	75	1	

**Eclampsia**				
Yes	8	9	2.17(0.412-11.47)	0.36
No	320	84	1	

**Antepartum haemorrhage **				
Yes	15	20	1.33(0.32-5.56)	0.691
No	313	73	1	

**Fetal presentation **				
Vertex	309	68	1	
Non-vertex	19	25	2.23(0.75-6.62)	0.148

**Mode of delivery **				
Vaginal	304	52	1	
cesarean section	24	41	6.97(2.87-16.93)	0

**Maternal conditions during labour and delivery**				

**Premature rupture of membrane**				
Yes	26	19	1.69(0.61-4.65)	0.308
No	302	74	1	

**Prolonged labour**				
Yes	22	36	3.33(1.32-8.38)	0.01
No	306	57	1	

**Cephalopelvic disproportion**				
Yes	6	15	2.18(0.38-12.34)	0.377
No	322	78	1	

**Peripartum pyrexia **				
Yes	8	11	3.72(0.93-14.81)	0.062
No	320	82	1	

**Neonatal conditions after delivery **				

**Meconium stained **				
Yes	39	55	8.55(4.20-17.39)	0
No	289	38	1	

**Prolapsed cord**				
Yes	10	11	3.58(0.95-13.53)	0.059
No	318	82	1	

**Prematurity **				
Yes	6	10	1.15(0.27-4.86)	0.841
No	322	83	1	

**Weight of neonate**				
<2.5kg	10	16	12.75(4.05-40.08)	0
2.5-4kg	310	72	1	
>4kg	8	5	0.31(0.028-3.62)	0.357

## Data Availability

The data used to support the findings of this study are available from the corresponding author upon request.
